# Genetic Analyses of Interactions among Gibberellin, Abscisic Acid, and Brassinosteroids in the Control of Flowering Time in *Arabidopsis thaliana*

**DOI:** 10.1371/journal.pone.0014012

**Published:** 2010-11-17

**Authors:** Malgorzata A. Domagalska, Elzbieta Sarnowska, Ferenc Nagy, Seth J. Davis

**Affiliations:** 1 Max Planck Institute for Plant Breeding Research, Cologne, Germany; 2 Institute of Plant Biology, Biological Research Centre, Hungarian Academy of Science, Szeged, Hungary; 3 School of Biological Sciences, Institute of Molecular Plant Science, University of Edinburgh, Edinburgh, United Kingdom; Temasek Life Sciences Laboratory, Singapore

## Abstract

**Background:**

Genetic interactions between phytohormones in the control of flowering time in *Arabidopsis thaliana* have not been extensively studied. Three phytohormones have been individually connected to the floral-timing program. The inductive function of gibberellins (GAs) is the most documented. Abscisic acid (ABA) has been demonstrated to delay flowering. Finally, the promotive role of brassinosteroids (BRs) has been established. It has been reported that for many physiological processes, hormone pathways interact to ensure an appropriate biological response.

**Methodology:**

We tested possible genetic interactions between GA-, ABA-, and BR-dependent pathways in the control of the transition to flowering. For this, single and double mutants deficient in the biosynthesis of GAs, ABA, and BRs were used to assess the effect of hormone deficiency on the timing of floral transition. Also, plants that over-express genes encoding rate-limiting enzymes in each biosynthetic pathway were generated and the flowering time of these lines was investigated.

**Conclusions:**

Loss-of-function studies revealed a complex relationship between GAs and ABA, and between ABA and BRs, and suggested a cross-regulatory relation between GAs to BRs. Gain-of-function studies revealed that GAs were clearly limiting in their sufficiency of action, whereas increases in BRs and ABA led to a more modest phenotypic effect on floral timing. We conclude from our genetic tests that the effects of GA, ABA, and BR on timing of floral induction are only in partially coordinated action.

## Introduction

Flowering is a critical phase transition in the development of angiosperms. The correct timing of this transition, such as it occurs under most favorable conditions, is essential factor determining reproductive success. The floral transition is an integrated response to various signal states of the plant [1]. The molecular mechanism of the control of flowering time has been most extensively studied in the model species *Arabidopsis thaliana* (Arabidopsis). An initial genetic survey with late-flowering mutants led to defining inductive photoperiods, extended exposure to cold, and the gibberellins (GAs) class of plant hormones phytohormones as major factors promoting flowering in Arabidopsis [2]. Further studies identified the effect of light quality, ambient temperature, stress, and other phytohormones in the flowering-time regulation [3].

Plant growth is synchronized by an array of phytohormones, which differentially affect multiple physiological, metabolic, and cellular processes, resulting in a coordinated developmental program. Known phytohomones include cytokinins, auxins, GAs, abscisic acid (ABA), brassinosteroids (BRs), and ethylene [4]. We note that various phytohormones have been implicated in regulating the floral transition [5]. As for example, the importance of GAs in the control of flowering time in Arabidopsis was first reported by Langridge in 1957, who showed that exogenous application of GAs hastened developmental timing [6].

In Arabidopsis, genetic and pharmacological experiments implicate GAs as promoters of flowering, particularly under non-inductive short-day conditions. One key experiment was the demonstration that *gibberellin deficient1* (*ga1*), a mutant blocked in biosynthesis of GA, was found to be delayed in flowering [7]. The mutant *gibberellin insensitive* [*gai*] defective in GA signaling is also delayed in the floral transition [8]. Reciprocally, mutants with enhanced GA-signaling, such as *spindly* (*spy*) and plants over-expressing *FLOWERING PROMOTIVE FACTOR1* (*FPF1*), which is believed to be involved in GA-signal transduction, flower early [9], [10]. Transgenic approaches to increase the level of endogenous GAs, caused by overexpression of the GA20 oxidase *GA5*, leads to a similar early flowering-time phenotype as GA application, particularly under short-day growth [11], [12]. Finally, double-mutant analyses with known late-flowering mutants revealed that the GA pathway is distinctive from other flowering-regulating pathways and that its activity is important during growth under a non-inductive photoperiod [2], [13].

The role of ABA in regulating the floral transition was initially proposed based on the early-flowering phenotype of an ABA-deficient mutant, indicating that ABA inhibits flowering [14]. In a study that has since been retracted, ABA was proposed to influence floral transition by direct binding to RNA-binding protein FCA [15], [16]. Whereas there is affirmative data that FCA does not directly bind ABA [17], [18], the core of this retracted manuscript could be correct. Notably, this work by Razem et al. clearly demonstrated the genetic and pharmacological effect of ABA on flowering time in Arabidopsis, and that this hormone delays flowering through up-regulation of the potent floral repressor *FLOWERING LOCUS C (FLC*). This non-controversial portion of that work (note that Figures 3 and 4 of the 2006 paper where not part of the 2008 retraction) indicates that ABA, at least in part, modulates flowering by affecting the transcript level *FLC* [15]. Interestingly, an independent study has demonstrated the inhibiting role of ABA on flowering time through modulating DELLA activity [19]. Collectively, one can infer that ABA is a floral repressor.

The promotive role of BRs in floral transition was proposed based on the late-flowering phenotype of BR-deficient mutants, *det2* and *dwf4* [20], [21], and early flowering of the *bas1 sob7* double mutant, which is impaired in metabolizing BRs to their inactive forms [22]. The finding that a mutation in the BR receptor *BRI1* leads to late flowering further supports the positive effect of BRs on the timing of floral transition. Interestingly, BR signaling also interacts with the autonomous pathway, as combining *bri1* with late-flowering autonomous mutants *ld* and *fca* results in delayed floral transition [23]. This late flowering is accompanied with an increase in expression of the floral repressor *FLC* in these double mutants [23]. This is consistent with observations that BR signals work within a chromatin pathway which requires ELF6 and REF6 as components in the floral-transition [24]. Thus, BRs are floral promoters.

It has been reported that for many physiological processes, hormone-signaling pathways do not function as separate entities. These pathways interact at various levels within the signaling process to ensure an appropriate biological response (reviewed in [25]). A well-described example of such hormone interactions is the regulation of seed germination, in which GAs and BRs have been shown to function antagonistically to ABA to break dormancy and promote germination [26]. We thus hypothesized that these three hormones might genetically interact in the regulation of the floral transition. This hypothesis seemed to be particularly attractive as both ABA and BRs signaling are proposed to interact with the autonomous pathway to modulate the levels of FLC in the control of floral transition [15], [23], and at the same time, salt (which activates ABA signaling) reduces levels of bioactive GAs [19].

In this work, we examined the possibility of genetic interactions between the GA-, the ABA- and BR-regulated pathways in the control of the transition from vegetative to reproductive development. The impact of mutations in the GA, ABA, and BR biosynthetic pathways was directly tested to assess their interactive network. Double-mutant combinations defective in the biosynthesis of GA, ABA, and BR were constructed and their flowering time was measured. Also, plants that over-express genes encoding rate-limiting enzymes in biosynthesis of GA, ABA, or BR were generated and their flowering time was investigated. We found that the hormone pathways tested appear to be complex in their promotive and repressive roles Furthermore, there appears to be a cross-regulatory effect between GA and BR signals.

## Results

### Analyses of genetic interactions between the *ga1, cpd*, and *aba2* mutants in flowering

**Figure 1:**
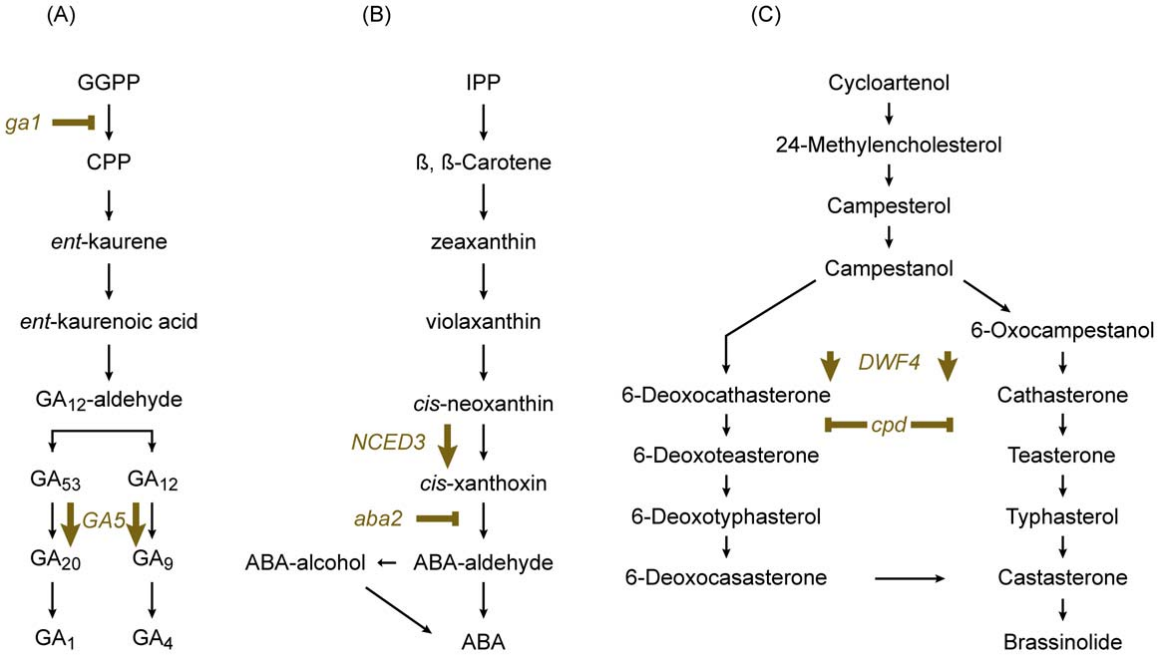
Simplified hormone biosynthetic pathways. The hormone biosynthetic pathways of Arabidopsis for gibberellins ***A.***, ABA ***B.***, and, brassinolide ***C.***. The biosynthesis mutants used in this study and sites of their lesions are shown. Also, the biosynthetic genes over-expressed to increase the levels of respective hormones are indicated. ***A.*** The *ga1* mutant is impaired in the first stage of GA-biosynthesis: the cyclization of geranylgeranyl diphosphate (GGPP) to copalyl diphosphate (CPP). ***B.*** The *aba2* mutant is blocked at the cis-xanthoxin to ABA-aldehyde conversion. ***C.*** The conversion of 6-Deoxocathasterone/Cathasterone to 6- Deoxoteasterone/teasterone does not occur in the *cpd* mutant. ***A.*** The *GA5* gene encodes a GA 20-oxidase that catalyzes the formation of the GA20 and GA9, the final precursors of the bioactive GAs. ***B.*** The *NCED3* encodes 9-*cis*-epoxycarotenoid dioxygenase that catalyzes the oxidative cleavage of a 9-*cis* isomer of epoxycarotenoid (9-*cis*-violaxanthin or 9’-*cis*-neoxanthin) to form xanthoxin. ***C.*** The *DWF4* gene encodes a 22-a hydroxylase (CYP90B1) that catalyzes the conversion of 6- Oxocampestanol/Campestanol to 6-Deoxocathasterone/Cathasterone. IPP, Isopentenyl pyrophosphate. ABA, abscisic acid. Adapted from [49].

**Figure 2:**
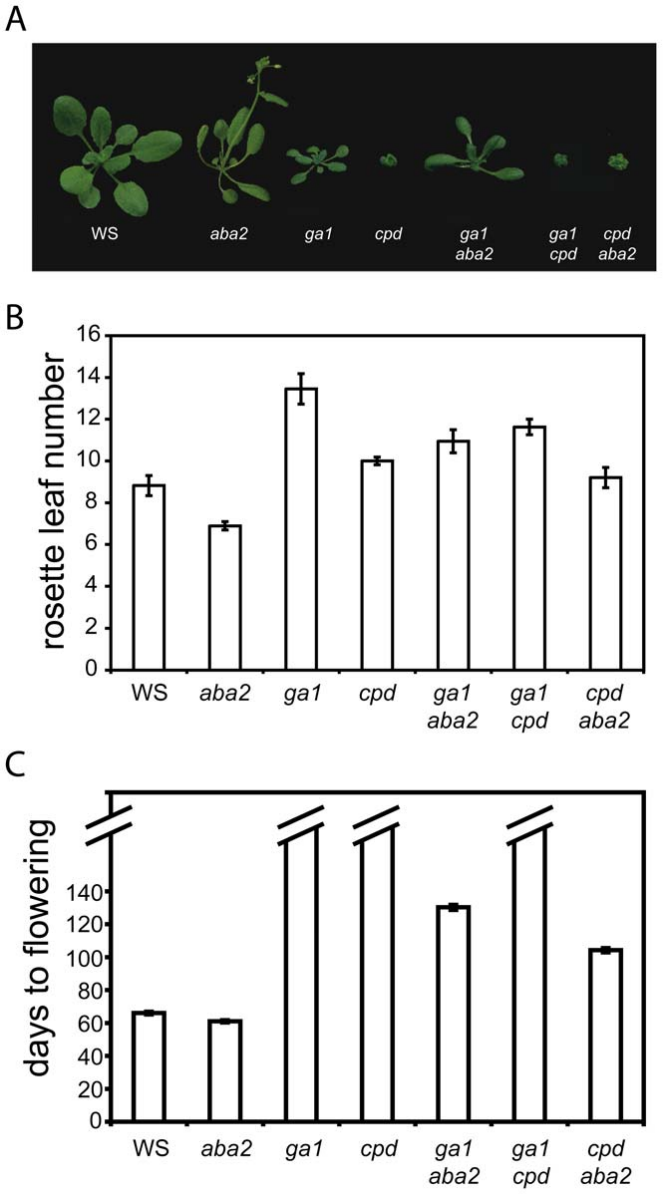
Floral-timing phenotypes of phytohormone mutants. ***A.*** Floral-timing phenotypes of the wild-type WS, the single *aba2*, *ga1, and cpd* mutants and the *ga1 aba2*, *ga1 cpd*, and *cpd aba2* double mutants. Plants were grown under long days (16 h light/8 h darkness) in controlled greenhouse conditions. Pictures were taken when wild-type plants were flowering. ***B.*** Flowering-time analyses of the wild-type WS, the single *aba2, ga1, and cpd* mutants and the *ga1 aba2*, *ga1 cpd*, and the *cpd aba2* double mutants. Plants were grown under long days (16 h light/8 h darkness) in the greenhouse. ***C.*** As in ***B.***, except plants were grown under short days (8 h light/16 h darkness) in the greenhouse. Flowering time was measured as rosette leaf number at bolting for ***B.*** or days to flowering for ***C***. Around 12 plants were scored per genotype. The hatched bars denote genotypes that did not flower over duration of measurement. Error bars represent SE. Two experiments were performed, and a representative result is shown.

To test for hormonal interaction in the control of the floral transition in Arabidopsis, we focused on potential relations amongst three known phytohormones: GAs, ABA, and BRs. To assess the interaction amongst them, we examined the effect of simultaneous reduction in the endogenous levels of two hormones, in all possible combinations. This was achieved by taking advantage of the existing hormonal-biosynthetic mutants *constitutive photomorphogenesis and dwarfism* (*cpd*), *gibberellin deficient1* (*ga1*), and *abscisic acid deficient2* (*aba2*) [27], [28], [29], [30]. The chosen *cpd, ga1*, and *aba2* mutants are blocked in the biosynthesis of BRs, GAs, or ABA, respectively (Fig. 1), and each exhibits deficiency phenotypes specific for the respective hormone. The morphology of these lines can be seen (Fig. 2A).

The double mutants (*aba2 ga1, ga1 cpd, aba2 cpd*) together with single *ga1, aba2, cpd* mutants, and the wild-type control, were subjected to flowering-time analyses under long- and short-day conditions. All single mutants in respective phytohormone pathways, under long days, flowered as expected when compared to the literature [27], [28], [29], [30]. In our studies, we confirmed previously reported phenotypes, namely, the *cpd* and *ga1* mutants being slightly late flowering, and the *aba2* mutant exhibiting modest early flowering. (Fig. 2B, Table 1). To assess potential genetic interactions, the pair-wise comparisons for each genotype to wild type, or to respective single mutants, were carried out. The double *aba2 ga1* mutant exhibited intermediate flowering phenotype between *ga1* and *aba2*, suggesting a lack of genetic interaction between these two hormonal pathways in the control of timing of the floral transition (Fig. 2B and Table 1). The phenotype of *aba2 cpd* double mutant was not significantly different from the single *cpd*, or the wild type (Fig. 2B and Table 1). This indicates that these two hormonal pathways act largely independently in the control of floral transition. In contrast, the double *cpd ga1* mutant flowered slightly later than the single *cpd* mutant, and this response was not different from the single *ga1* under the experimental conditions tested (Fig. 2B and Table 1).

**Table 1:** Student's t-test for flowering-time differences between mutant genotypes.

Genotype 1	Genotype 2	P value
WS	*aba2*	0.003123 *
WS	*cpd*	0.026681 *
WS	*ga1*	0.000035 ***
WS	*aba2 cpd*	0.638560 ø
WS	*aba2 ga1*	0.012466 *
WS	*cpd ga1*	0.000483 **
*aba2*	*aba2 cpd*	0.000231 *
*cpd*	*aba2 cpd*	0.068565 ø
*aba2*	*aba2 ga1*	0.000025 ***
*ga1*	*aba2 ga1*	0.009909 *
*cpd*	*cpd ga1*	0.000359 **
*ga1*	*cpd ga1*	0.063013 ø

Listed are pairs of compared genotypes. P values for each pair are provided.

ø No significant difference P>0.05;

statistically significant differences:

***P<0.0001,

**P<0.001,

*P<0.05.

We next examined the timing of flowering in phytohormone-biosynthetic mutant combinations under non-inductive short-day conditions. Late-flowering genotypes grown under non-inductive photoperiods result in plants that had leaf senescence before bolting occurred (data not shown). Thus, leaves were "missing" by the time bolting commenced. Furthermore, the morphology of several mutant combinations precluded accurate leaf counting. For these reasons, we scored the number of days to bolting as a direct measure of flowering time for these short-day experiments. In these experiments, the *ga1* mutant did not flower during the extended duration of growth (Fig. 2C). Non-flowering responses were observed in the *cpd* and the double *ga1 cpd* mutants. The *aba2* single mutant flowered slightly earlier than wild type. (Fig. 2C). Furthermore, the reduction in endogenous ABA levels due to a lesion in *ABA2* led to both the *ga1* and the *cpd* mutants to flower within the duration of the assay, in their respective double mutants (Fig. 2C). With an analysis using Student's t-test, all genotypes were statistically separable in all pair-wise combinations (P<0.002). Taken together, complex interactions resulted when examining the reduction of GAs, ABA, and BRs, when considering the timing of flowering under inductive long days and non-inductive short-days.

### Flowering-time analyses of plants with elevated expression of rate-limiting enzymes in the biosynthesis of GAs, ABA, and BRs

**Figure 3:**
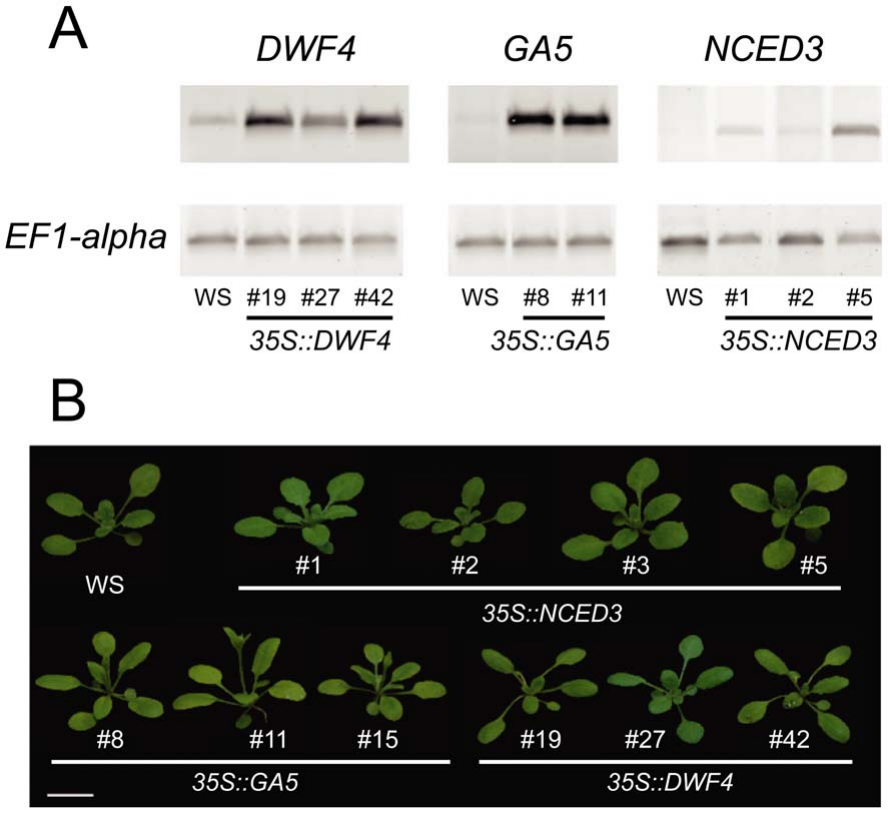
Overexpression lines for rate-limiting enzymes in various phytohormone pathways. Transgenic lines harboring *35S::DWF4*, *35S::GA5* and *35S::NCED3* constructs. ***A.*** Over-expression was confirmed by RT-PCR with primers specific for *DWF4*, *GA5* and *NCED3*. Primers specific for the elongation factor 1-alpha gene were used as a control. Representative lines are shown. All lines tested showed over-expression of the gene of interest >3 fold. ***B.*** Images of 3-weeks-old plants grown under long days (16 h light/8 h darkness) in the greenhouse. The white bar indicates 1 cm.

To further examine the role of GAs, ABA, and BRs in the floral transition, we analyzed the effect of elevated endogenous levels of each hormone on flowering time under long- and short-day growth conditions. Transgenic plants over-expressing rate-limiting enzymes in BR, GAs, and ABA biosynthesis were generated. For this, respectively, the *DWF4, GA5*, and *NCED3* genes were chosen. Their relative positions in respective biosynthetic pathways are depicted in Fig. 1. These genes have been previously shown to cause an increase in the endogenous levels of respective hormone or its precursor when over-expressed [11], [12], [31], [32]. These selected genes were expressed under control of the Cauliflower Mosaic Virus 35S promoter, which enabled their expression to high levels. The over-expression of the genes of interest was confirmed using RT-PCR with gene-specific primers (Fig. 3A), and further, the levels of reaction products were quantified. All transcript levels were found for all lines to be >3 fold increased, compared to the wild type (data not shown). Furthermore, the obtained transgenic lines displayed morphological and physiological phenotypes attributed to the overproduction of the respective hormones, as described in respective previous reports [11], [12], [31], [32]. We concluded that these lines were suitable for flowering-time studies.

**Figure 4:**
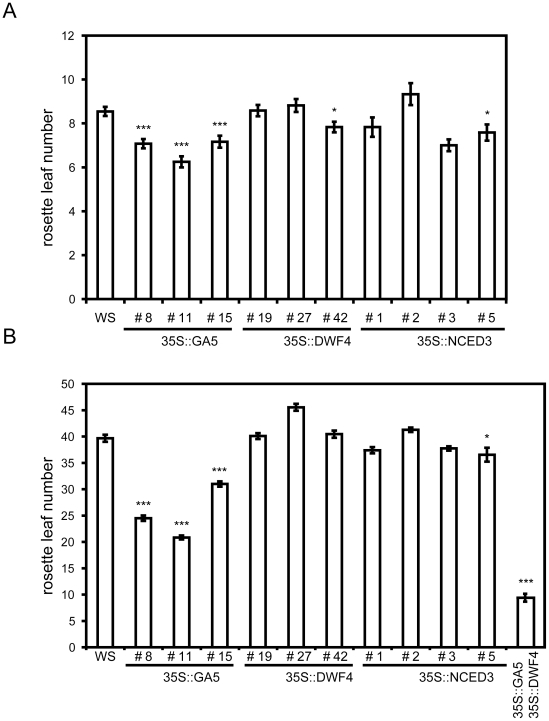
Floral-timing phenotypes of phytohormone overexpression lines. Flowering time of the transgenic lines that over-express GA-, BR- and ABA-biosynthetic genes: *GA5, DWF4* and *NCED3*, respectively. ***A.*** Long-day conditions (16 h light/8 h darkness). ***B.*** Short-day conditions (8 h light/16 h darkness). Flowering time was measured as rosette leaf number at bolting. Around 12 plants were scored per genotype. Error bars represent SE. Student's t-test was applied to test for the differences in flowering time, relative to the wild type, P<0.0001***, P<0.05*.

The *35S::DWF4*, *35S::GA5*, and *35S::NCED3* lines were subjected to flowering-time analyses under long- and short-day growth conditions (Fig. 4A, B). The flowering time of similar *35S::GA5* genotypes has already been reported [11], [12], and the results described here are therefore confirmatory. The differences in flowering times amongst genotypes were compared with an analysis using Student's t-test. As expected, three representative lines of the *35S::GA5* flowered early under both long and short days (P<0.0001). Neither *35S::DWF4* nor *35S::NCED3* exhibited a consistently altered flowering time. Under long days, only one out of three *35S::DWF4* lines flowered marginally early (line #42, P<0.05). Under short days, none of the lines displayed reproducible changes in flowering time. The *35S::NCED3* line #5 was the only one out of four *35S::NCED3* lines that displayed marginally accelerated flowering in a reproducible and significant manner (P<0.05), under both photoperiods of tested growth. Hence, whereas GAs had a clear concentration-limiting role in the flowering-time control, ABA and BR do not seem to be limiting in a concentration-dependent manner for timing of floral transition.

In the double-mutant analysis, we observed that *ga1* and *cpd* generated late flowering, and that *ga1* could enhance the *cpd* phenotype (Figure 2). This could suggest that in the absence of BRs, the additional absence of GAs leads to a maximal hormone block in the generation of late flowering phenotype. In this sense, *ga1* would be epistatic to *cpd*; no additive effect was detected in the *ga1 cpd* double mutant (Figure 2). We hypothesized that the promotive effects of BRs would only be observed in the presence of increased GAs levels. To test this, the double *35S::DWF4/35S::GA5* transgenic line was generated, and this genotype was analyzed for its flowering time under non-inductive short-day conditions. Consistent with this hypothesis, the double *35S::DWF4/35S::GA5* flowered significantly earlier than the single *35S::GA5* line (P<0.001). (Fig. 4B). This result clearly demonstrates a major rate-limiting role of GAs in floral promotion. It also implies that BRs' promotive role in the transition to flowering depends on the presence and concentration of GAs.

## Discussion

Previous analysis of the individual hormonal effects of GAs, ABA, and BRs have supported that each has a role in the transition from vegetative to reproductive development. Here we examined whether these effects had any interdependence. Using loss-of-function and gain-of-function studies, we were able to conclude that genetic interactions between these hormone-pathways in reproductive timing were complex. Further, whereas the genetic depletion of any of the three tested hormones led to timing defects, for genetically increased levels of hormones, only GA led to noted physiological timing defects; the sole increase of ABA and BR did not lead to dramatically modified responses. As an example of the complexities, BR effects where most noted in the context of a transgenic that also was increased for GA. Taken jointly, there was clearly a dominant role of GAs as the phytohormone that promotes the transition from vegetative to reproductive development.

The analyses of the flowering phenotypes of double *aba2/ga1/cpd* mutant combinations revealed the basis of their genetic interactions (Fig. 2, Table 1). Based on the flowering behavior of the double *aba2 ga1* mutant, compared to the respective single mutants, we concluded that the block in ABA and GA synthesis, respectively, result in independent phenotypic effects on flowering time. We note that others have reported a direct cross-regulatory interaction between ABA and GA hormonal pathways with the discovery that a component of the ABA biosynthesis pathway, and in drought tolerance, where a direct target for GA action *via* the so-called DELLA proteins [33]. From there, we further found no significant difference under inductive photoperiods for the flowering time between the double *cpd aba2* and single *cpd* mutants, which suggested to us that the BR-deficient mutant is epistatic to the ABA-biosynthesis mutant, at least under examined conditions. As well, since the double *cpd aba2* did not differ from wild type, we interpreted this as that the phenotypic effect generated by the *aba2* mutation was different from that resultant from the *cpd* mutation. We cannot exclude that the circadian effects on the photoperiod pathway generated from BR and ABA signaling are not canceling out, as these hormones have opposite effects on the "speed" of clock periodicity [34].

The statistical difference between the *cpd ga1* double mutant and the *ga1* single mutant under long-day conditions is genetic support that BR- and GA-pathways genetically interact and/or that GA levels are modified by the genetics of BRs, as has been shown previously [26], [35]. Furthermore, it appears that *ga*- and *br*-synthesis mutants can cause cross-regulatory effects on the reciprocal hormone homeostasis levels [35], [36], [37]. Although, this is not always the case [38]. Taken together, the relationships between the studied hormonal pathways in the control of flowering time are concluded to be complex and the genetic relations of these three pathways cannot be put into a simple linear pathway. In contrast, it appears that there are cross-regulatory mechanisms that function on several levels. Similar responses have also been reported by others [39]. A part of the genetic complexity could be caused by reciprocal, differential regulation of the hormone biosynthetic genes by various hormone-signaling pathways, as it has been shown that in seedlings BR and GA antagonistically regulate the accumulation of mRNAs of the GA-regulated *GASA1* and *GA5* genes [35].

ABA increases were not found to generate large effects on floral timing. Transgenic lines that overexpressed the *NCED3* genes did not exhibit strong flowering phenotypes (Fig. 3, 4). In general, *35S::NCED3* plants were slightly earlier flowering than wild type, except one line that was marginally delayed in flowering. Those effects were not statistically significant. Our results trended differently from what has been published recently regarding the effect of pharmacological manipulation of ABA on the floral transition. For instance, it has been reported that exogenous ABA delays flowering, and that this correlated with the up-regulation of *FLC* [19], [40]. As mentioned earlier, the *35S::NCED3* plants exhibited increased expression of *NCED3* and an ABA-over-expression phenotype, including delayed germination and growth, and activation of some ABA-regulated genes [32]. We also observed such effects (Fig. 3B and data not shown). It has also been shown that over-expression of this ABA-biosynthetic gene results in an elevation of the endogenous levels of ABA. Thus, the lack of a strong phenotype in the generated *35S::NCED3* plants was under a context of increased ABA content. Perhaps the endogenous levels of ABA in plants overexpressing *NCED3* were lower compared to ABA levels obtained through exogenous application of ABA reported [15], [19] (we note that they reported that a significant delay in flowering was not observed with the addition of 1 µM ABA, and was only with a pharmacological level of 10 µM ABA was an effect seen).

It has been considered that ABA is a "stress hormone," because its levels increase upon stress treatment. Furthermore, it mediates the response to drought and other stresses [41]. It has also been reported that drought accelerates flowering [42]. Hence, we wonder if at low concentrations ABA inhibits flowering, and after reaching a certain threshold, it induces the floral transition. This could explain the mild early flowering that can be observed in some *35S::NCED3* lines. Further detailed studies on the mechanism of drought- and ABA-induced flowering are required to resolve this issue.

As with ABA, increases in BRs did not dramatically alter the wild type for floral-promotive effects. No pronounced flowering phenotype was detected when *35S::DWF4* lines were analyzed (Fig 3, 4). Under long days, only one line was found to flower statistically earlier, and therefore, overproduction of BRs seems not to affect flowering under this condition. Under short days, only one of three *35S::DWF4* lines displayed mild later flowering. Thus, BRs may not have a rate-limiting role in floral promotion.

In support of previous findings [11], transgenic efforts to increase endogenous GA pools caused accelerated flowering time (Fig. 3, 4). The *35S::GA5* plants we generated clearly flowered earlier under both photoperiodic conditions tested, confirming the importance of GAs in the control of the transition to flowering. Given the apparent redundancy of the *cpd* phenotype on the *ga1* phenotype (Table 1), and the clear action of *35S::GA5* on promoting flowering, and the lack of clear effect *35S::DWF4* on promoting flowering (Fig. 4), it was of interest that *35S::DWF4* introduction accelerated the floral transition in the context of the *35S::GA5* (Fig. 4B). One interpretation is that GA is limiting to promote flowering in the context of elevated BR levels. Collectively, of all transgenic up-regulation responses tested, GA elevation had the most marked effect. This supports the notion that GA is a dose-dependent regulator of the reproductive transition.

The phenotype of the BR- and ABA-deficient mutant and the lack of a significant flowering phenotype in the transgenic lines that over-express the *DWF4* and *NCED3* genes, leads us to a conclusion that these hormonal pathways are necessary for proper timing of the floral transition, but are themselves insufficient to significantly modify the transition time. GA in turn, seems to be a "master" hormone over ABA/BRs. This hypothesis is furthered by the clear late-flowering effect of the *ga1* mutation, particularly under non-inductive photoperiods [7]. The over-expression of the *GA5* gene resulted in a clear early-flowering phenotype, regardless of the photoperiod, confirming the promotive role of this hormone. Finally, the dominant role of gibberellins, followed by a supporting function of ABA and BRs can be inferred from the analyses of the double hormonal mutants. Collectively, we report that hormone regulation on the transition from vegetative to reproductive development depends on an overall balance of GAs, ABA, and BRs.

## Materials and Methods

### Plant material

Experiments were carried out using *Arabidopsis thaliana* ecotype *Wassilewskija-2*, termed in the paper WS. The *ga1-3* mutant, originally in the L*er* background, was backcrossed into WS, as described in [23]. *cpd-3939* was a gift from F. Tax (University of Arizona) [23], [43] and *aba2-2* (*gin1–1*) was kindly provided by J. Sheen (Harvard University) [29]. Single *cpd*, *aba2*, and *ga1* mutants were crossed to each other in order to obtain double mutants. The resultant double mutants were isolated by identifying homozygous lines for *aba2*, and *ga1* mutation, based on glucose-insensitivity and GA-deficiency, respectively [7], [29]. Plants heterozygous for *cpd* were found in the F3 generation by identifying dwarf "cabbage"-looking plants. Since the *cpd* mutant is male sterile, the double homozygous mutants were always visually selected from the segregating population during each experiment. To isolate the *aba2 ga1* double mutant, the selected in the F2 generation GA-deficient mutants were self-fertilized and in the next generation lines homozygous for the *aba2* mutation were isolated with the previously described molecular marker [29]. Identified in this way the *aba2 ga1* mutant was self-fertilized and its progeny was used in further experiments.

To construct plants over-expressing *DWF4, NCED3, GA5* genomic clones were amplified with primer pairs:

*DWF4* with (GWF)CCATGTTCGAAACAGAGCATCA and (GWR)TTACAGAATACGAGAAACCCTAATA, *GA5* with (GWF)CCATGGCTTCTTTCACGGCAACG and (GWR)TCACACGACCTGCTTCGCCA, and *NCED3* with (GWF)CCATGGCCGTAAGTTTCGTAACAA and (GWR)TTAGATGGGTTTGGTGAGCCAA. GWF denotes GGGG*att*B1 site, GWR denotes GGGG*att*B2 site, (GATEWAY®, Invitrogen, Germany). Purified PCR-products were separately inserted into the pDONR207 vector by means of BP reaction (GATEWAY®, Invitrogen, Karlsruhe, Germany). The accuracy of cloned gene sequences was confirmed by sequencing. Subsequently, the cloned *DWF4, GA5, NCED3* genes were inserted downstream of the 35S promoter into the plant-transformation pLeela vector [44] using an LR reaction. The resulting constructs were transformed into *Agrobacterium tumefaciens* GV3101 pMP90RK strain [45], which was used to transform wild-type WS Arabidopsis plants by means of the improved floral-dip method [46]. Transgenic plants were selected based on their resistance to Basta, as described [47]. Plants were confirmed to harbor a transgene by genotyping with 35S-specific primers and gene-specific primer used for cloning. Plants were backcrossed to WS, and in F2 generation lines that harbored one insert (as judged by scoring the segregation of a single locus of resistance to Basta) were used for further experiments. Homozygous lines, resultant from such transgenic lines, were those used for experimentation. The double *35S::DWF4/35S::GA5* transgenic line was generated by crossing the relevant single transgenics and selecting in the F2 and F3 generations the required genotype.

### Analysis of mRNA abundance

Transcript abundance was analyzed by reverse transcriptase (RT)-PCR, exactly as described [23]. Primers to amplify *EF1α* where GTTTCACATCAACATTGTGGTCATTGG and GAGTACTTGGGGGTAGTGGCATCC; primers to amplify *DWF4* were TCCCTAGTGGGTGGAAAGTG and TTACAGAATACGAGAAACCCT; primers to amplify *GA5* were AAGGCCTTTGTGGTCAATATCGGC and TTAGATGGGTTTGGTGAGCCAA; primers to amplify *NCED3* were CAAGATTCGGGATTTTAGACA and TCACACGACCTGCTTCGCCA. PCR products were separated on ∼2.5% agarose gels. The DNA was stained with ethidium bromide and photographically visualized. PCR products were visualized and analyzed for saturation levels using KODAK 3 system. For the densitometry measurement, Image J 1.42 software was used [48].

### Plant growth condition and flowering time experiment

Experiments were conducted similarly as described [23]. Briefly, seeds were stratified for 2–5 days at 4°C in darkness on half-strength MS-medium without sucrose (Sigma-Aldrich, Taufkirchen, Germany), with 1.2% (w/v) agar or MS-medium without sucrose supplemented with 50 µM GA_3_, followed by 1–2 days incubation under the light (long-day photoperiod), prior to transferring to soil. Flowering-time experiments were performed in a temperature- and photoperiod-controlled greenhouse and in climate-controlled growth chambers. The long day consisted of 16 hours of light, followed by 8 hours of darkness; the light intensity was 80–160 µmol s^−1^ m^−2^. The short day-condition consisted of 8 hours of light and 16 hours of darkness, the light intensity was 100–150 µmol s^−1^ m^−2^; the temperature was ∼22°C. Approximately twelve plants per genotype were analyzed in each experiment. Standard error (SE) was measured. Experiment replications provided similar results. Flowering time was scored as the number of rosette leaves at flowering, or days to bolting, when the bolt was *ca.* 1 cm high.
